# Design and biological evaluation of novel BF-30 analogs for the treatment of malignant melanoma

**DOI:** 10.7150/jca.47549

**Published:** 2020-10-18

**Authors:** Jia Qi, Weiwei Wang, Wenying Lu, Wei Chen, Hui Sun, Anquan Shang

**Affiliations:** 1 Department of dermatology, Nanjing Medical University Affiliated Wuxi Second hospital, Wuxi, Jiangsu, 214002, China.; 2 Department of Laboratory Medicine, The Sixth People's hospital of Yancheng City, Yancheng, 224001, Jiangsu, China.; 3 Department of Laboratory Medicine, Tongji hospital of Tongji University, Shanghai 200065, Shanghai, China.

**Keywords:** Cathelicidin-BF, controlled-release, albumin binding, thrombin, melanoma

## Abstract

**Aims:** To evaluate anti-tumour effects and mechanism of novel BF-30 derivative via cell-based assays and melanoma-bearing model mice.

**Main methods:** BF-30 derivatives were designed by fusing heptapeptide-palmitic tags to native BF-30 via a protease-cleavable linker and prepared by F-moc solid-phase synthesis. Albumin binding affinity test and in vitro control-released assay were performed to screen these BF-30 derivatives and explore the mechanism of anti-tumour effects. The selected BF-30 derivative was further subjected to the preclinical efficacy study and chronic evaluation of anti-tumour effects melanoma-bearing model mice.

**Key findings:** Twenty-one BF-30 derivatives, termed LBF-1 to LBF-21, were obtained with high purity and accurate molecular weight. Surface plasmon resonance (SPR) measurements, plasma stability test and in vitro control-released assay all showed that LBF-14 exerted better druggability compared with the others. Moreover, LBF-14 was proved to inhibit the proliferation of B16F10 melanoma cell by disrupting the cytoplasmic membrane and binding to genomic DNA to prevent transcription. Furthermore, half-life of intact LBF-14 and released BF-30 in rhesus monkeys were approximately 120.9 h and 136.4 h, respectively, after a single subcutaneous injection of 0.9 mg/kg LBF-14. In addition, chronic treatment of LBF-14 significantly suppressed melanoma growth and improved the survival rate of B16F10-bearing mice with the observed inhibition of 63.5% for 0.3mg/kg and 91.5% for 0.9 mg/kg. Furthermore, results of H&E staining prove that chronic treatment of LBF-30 effectively suppressed metastasis and invasion of B16F10 cells.

**Significance:** LBF-14 holds potential to be developed as a promising once-weekly candidate for the treatment of malignant melanoma.

## Introduction

Cathelicidin-BF (BF-30), as a 30-mer polypeptide isolated from *Bungarus fasciatus* serum with a secondary structure of amphipathic α-helical conformation, exhibits outstanding anti-microbial activities 1, 2. Previous researches revealed that BF-30 is a phenylalanine- and lysine-rich polypeptide exhibiting broad spectrum and strong antibacterial properties without drug resistance. Moreover, BF-30 exerted antibacterial acitivities through enhancing the permeability of cytoplasmic membrane which is superior to the common antibiotics 2. In addition, the cathelicidins, such as hCAP-18 and BMAP-28, were previously proved to be cytotoxic to numerous cancer cells including melanoma, breast, ovarian, leukemia, lung, lymphoma and cervical 3. Wang et al. previously demonstrated that native BF-30 may selectively inhibit the proliferation of melanoma cells via *in vitro* permeabilization of cytoplasmic membrane and DNA-binding, and exerted the outstanding anti-cancer effects in B16F10-bearing mice 4. However, native BF-30 was restricted in a short lifespan which mainly caused by the rapid glomerular filtration and protease digestion 5.

Consequently, plenty of strategies were developed to increase the therapeutic duration of BF-30* in vivo* including enlarging the molecular size via PEGylation to prevent from the rapid renal filtration and enzymatic degradation 6. However, the chemical PEGylation may hamper the therapeutic effects of the BF-30 or other peptides because the increased steric hindrance prevents the binding of targeting molecules 6. Coupling the therapeutic polypeptide to plasma protein, or protein domain which could bind to the plasma protein, shows a promising way to extend short half-lives* in vivo* 7, 8. Human serum albumin (HSA), as one of most useful *in vivo* molecule transporters, exerts an overlong *t*_1/2_ of ~19 days and unique advantages as a drug carrier to prolong the half-lives of therapeutic peptides 9, 10. However, direct fusion to HSA often reduces the bioactivities of polypeptides, which is similar to the PEGlyation 11. To regain peptide activity, the coupled peptide should be liberated as its fusion form without modification.

Based on previous study 9, we fused the therapeutic peptide with a lipidated the heptapeptide tags exerting high affinity for HSA to extend its short half-life. In order to develop a fully novel BF-30 with long-acting *in vivo* duration, we fused previously designed tags to native BF-30 via a protease sensitive sequence and obtained twenty-one BF-30 fusion peptides. The newly designed BF-30 derivatives could tightly associate to HSA and, theoretically, could be protected from rapid glomerular filtration and proteolytic digestion in blood circulation. Whereafter the thrombin recognizes and digests the protease cleavable sequence. As a result, the native BF-30 is then slowly released into the blood circulation.

In this study, we aimed to design, characterize and evaluate the novel long-acting BF-30 derivatives with peptidic albumin binding domain and prolonged in vivo duration for the cancer treatment. Necessary sequence and modification optimization were carefully performed via the *in vitro* assays including albumin binding affinity and plasma stability tests. Further preliminary *in vivo* pharmacodynamics and pharmacokinetics evaluation of BF-30 derivatives were also carefully studied.

## Materials and methods

### Materials and animals

All the peptides used in this research were obtained from GL biochem (Shanghai, China) by using a traditional solid phase synthesis method. HSA, methyl thiazolyl tetrazolium (MTT), dimethylsulfoxide (DMSO) and other reagents were acquired from Sigma-Aldrich (Merck, Germany). The cancer cell line used in this study was obtained from American Type Culture Collection (ATCC, USA). Fetal bovine serum, RPMI-1640, as well as F12 medium were all purchased from Gibco (Vienna, USA). The BF-30 ELISA kit was purchased from Nanjing Jiangche Biotechnology Co., Ltd (Nanjing, China).

The C57BL/6J mice (male, 6-8 weeks) and cynomolgus monkeys (male, 3-4 months) were purchased from JOINN Laboratories (Beijing, China). Mice were grouped in six/cage while the monkeys were in one/cage, and all the animals were housed in a humidity and temperature controlled room under a 12-h light-dark cycle. The experimental animal studies were performed in conformity to the Laboratory Animal Management Regulations in China and approved by JOINN Laboratories Animal Ethics Committee with the approval code of ATC-190234.

### Design, preparation and identification of LBF peptides

Peptides were synthesized according to the traditional strategy of F-moc solid-phase synthesis and the crude peptides were purified by HPLC (using the agilent C18 reversed-phase column; detection wavelength: 280 nm; flow rate: 0.5 ml/min; mobile phases: acetonitrile and deionized water) with all purities more over 98%. Accurate molecular weight of LBF peptides were identified using MALDITOF/MS (Bruker, Karlsruhe, Germany).

### Albumin binding affinity measurements

The binding affinities of LBF peptides for albumin were detected by surface plasmon resonance (SPR) measurement using BIAcore 8K (G&E, USA). Experimental details were conducted following the manufacturer's instructions. In general, the albumins were fixed to CM5 chip by using an amine coupling method. The successful immobilization of albumin was confirmed by the observation of a ~100 RU increase in the sensor chip. Then the LBF derivatives were further diluted to different gradients (0, 0.1, 0.3, 0.9, 2.7, 8.1, 24.3 nM) and consequently flew through CM5 chip with the albumins at a flow rate of 50 μL/min under a model of 300 s binding and 300 s dissociating in the assay buffer HBS-EP+ at 25℃. The binding sensor grams (RU versus time) were pooled, trimmed, double referenced, and experimentally fit to a model of 1:1 binding after double deduction by using BIAcore T200 (G&E, USA).

### *In vitro* release study

Protease cleavage tests were performed in PBS buffer (pH 7.4) containing LBF derivatives and thrombin at final concentration of 60 μM and 0.4 U.mL^-1^, respectively. The compounds were then incubated at 37°C for 7 days in the dark and the samples were taken out at day 0, 1, 2, 4 and 7, respectively. Finally, the different hydrolysed fragments including the released BF-30 were identified using the method of ELISA or LC-MS/MS.

### Plasma stability assay

Albumins in the mouse plasma were removed through a SwellGel Blue Albumin Removal Kit (Thermo Fisher Scientific, America) according to the manufacturer's instructions. LBF-13 to LBF-15 were incubated in mouse plasma with or without albumin at 37℃ for 7 days with initial concentration of 1 mg.L^-1^. Then the compounds were collected at various time points of day 0, 1, 2, 3, 4, 5, 6 and 7, then further detected by the developed LC-MS/MS method.

### Cell viability assay

In this study, the *in vitro* anti-tumour activities of LBF-14 against cancer cell line B16F10 were determined using the CCK-8 assay. Firstly, 50 μL of B16F10 cells at the concentration of 10^4^ cells/ml were inoculated into a 96-well plate overnight with RPMI-1640 medium containing 10% fetal calf serum, and 20 μL of enzymatic mixture of LBF-14 at different time were added into the wells for 6 and 24 h with three duplicates. Then, 20 µL of CCK8 was added to the wells. After incubation at 37℃ for 3 h, the absorbance at 450 nm was then measured using an enzyme-linked immunosorbent assay analyzer. The cell growth, indicating the magnitude of toxicity, was calculated according to previously reported equation 4.

### DNA retardation assay

The DNA retardation assay was performed to access the DNA binding ability of released BF-30 from enzymatic LBF-14 according to the previous reports 12. The genomic DNA of B16F10 cell were separated by the kit obtained from Sangon Biotechnology (Shanghai, China), the DNA concentration was measured by using a spectrophotometric method at 260 nm, and then of 5 μM genomic DNA were mixed with the released BF-30 from LBF-14 at day 1, 4 and 7 for half an hour. The migration of DNA bands was measured by the agarose gel electrophoresis and detected using UV illuminator. The quantification of DNA levels of different time was identified by density analysis using the Image J. software. In addition, DNA binding ratios (%) of these groups were calculated as [1-(A/B)]×100% (A: electrophoretic band; B: genomic DNA band).

### Pharmacokinetics test

The pharmacokinetics parameters of LBF-14 were further determined in cynomolgus monkeys. Following the single subcutaneous (s.c.) injection of LBF-14 (0.1, 0.3 and 0.9 mg/kg) in monkeys, the blood samples were collected at various time-pionts (0, 4, 8, 12, 24, 48, 72, 96, 120, 144, 168 and 192 h) from right fore. The plasma drug levels of free BF-30 were detected by using the commercial BF-30 ELISA kit, while that of intact LBF-14 were measured by using the established LC-MS/MS method.

### Chronic study in B16F10-bearing mouse model

The B16F10 cells (0.1 ml, 5×10^6^ cells, cell viability >99%) were subcutaneously transplanted into the C57BL/6J mice. In the next 15 days, the length (L), width (W) and height (H) of tumours were measured with a caliper to calculate tumour volume using the formula: V = π/6×L×W×H. When the tumours grew to 150-350 mm^3^, the melanoma model mice were randomly divided into five groups (n=10) and received saline, native BF-30 at 0.9 mg/kg, LBF-14 at three doses of 0.3 and 0.9 mg/kg, and cisplatinum at the dose of 0.1 mg/kg, respectively. All the samples including the saline and different doses of LBF-14 were administered twice weekly by intra-tumour injection (50 μl) for 4 successive weeks. All melanoma model mice were weighted and sacrificed at the end of the experimental period (day 29). The anti-tumour efficacies of LBF-14 at both two doses were presented as inhibitory rates (%) which were calculated as [(A-B)/A]×100% (average tumour weight of (A) saline treated group and (B) LBF-14 treated groups, respectively). In addition, the tumours from the B16F10-bearing mice were separated and then performed H&E staining according to the standard operating procedures.

### Statistical analysis

The data were analyzed using GraphPad Prism 5 (GraphPad Software Inc., San Diego, CA, USA) statistical package, all measured variables were presented by mean ± SD. Differences in all parameters were tested using one-way ANOVA. P values lower than 0.05 were considered significant.

## Results

### Design, preparation and identification of novel BF-30 analogs

**Figure 1:**
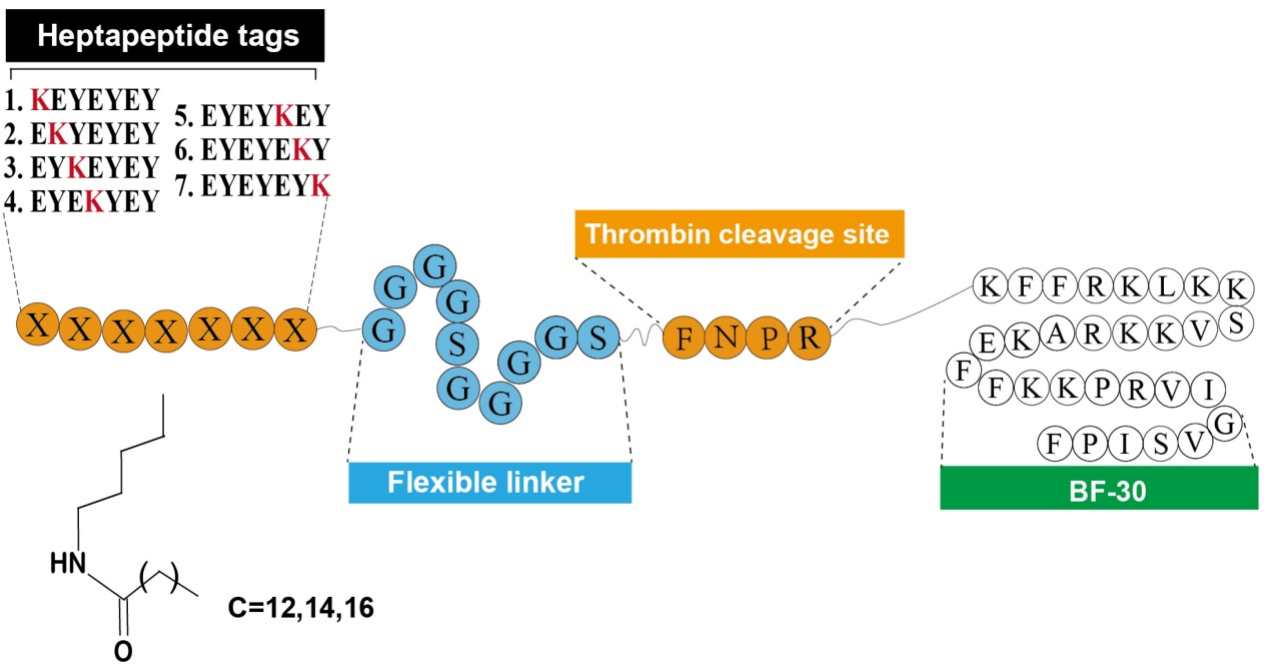
Schematic illustration and structure of LBF peptides.

As was reported previously 9, the heptapeptide-palmitic tags were identified with high affinity for HSA. As showed in Figure 1, using the BF-30 as the bioactive peptide, twenty-one hybrid peptides (termed LBF-1 to LBF-21) were designed by fusing the native BF-30 to seven heptapeptide-palmitic tags with different positions of lysine (KEYEEYE, EKYEEYE, EYKEEYE, EYEKEYE, EYEEKYE, EYEEYKE and EYEEYEK) and different length of aliphatic acids (C12, C14, C16) via a flexible linker (GGGGSGGGGS) and thrombin-cleavable site (FNPR). All the peptides were prepared by using the standard method of solid phase synthesis with the acute molecular weight and purities more than 98% (Table 1).

### Albumin binding affinity measurements

According to the above described design of LBF peptides, which hold the strong albumin-binding abilities, we further measured the related binding constants including the association rate constant (k_a_, 1/Ms), dissociation rate constant (k_d_, 1/s) and overall affinity constant (*K*_D_, M) of LBF-1 to LBF-21 by using the SPR method. As shown in Table 2, LBF-13 to -15 exerted albumin binding affinity for 2.43×10^-7^ M, 4.31×10^-7^ M and 3.04×10^-7^ M, respectively, which were higher than that of other conjugates. Above results suggested that the HSA binding affinity of LBF peptides were closely related to the position of site-specific modification in heptapeptide-palmitic tag and the fifth acylation site modified showed the highest potency to that of the others. Therefore, LBF-13 to -15 were selected to perform the further protease cleavage assay to determine the suitable length of aliphatic acid chain.

### *In vitro* release of BF-30 analogs

**Figure 2:**
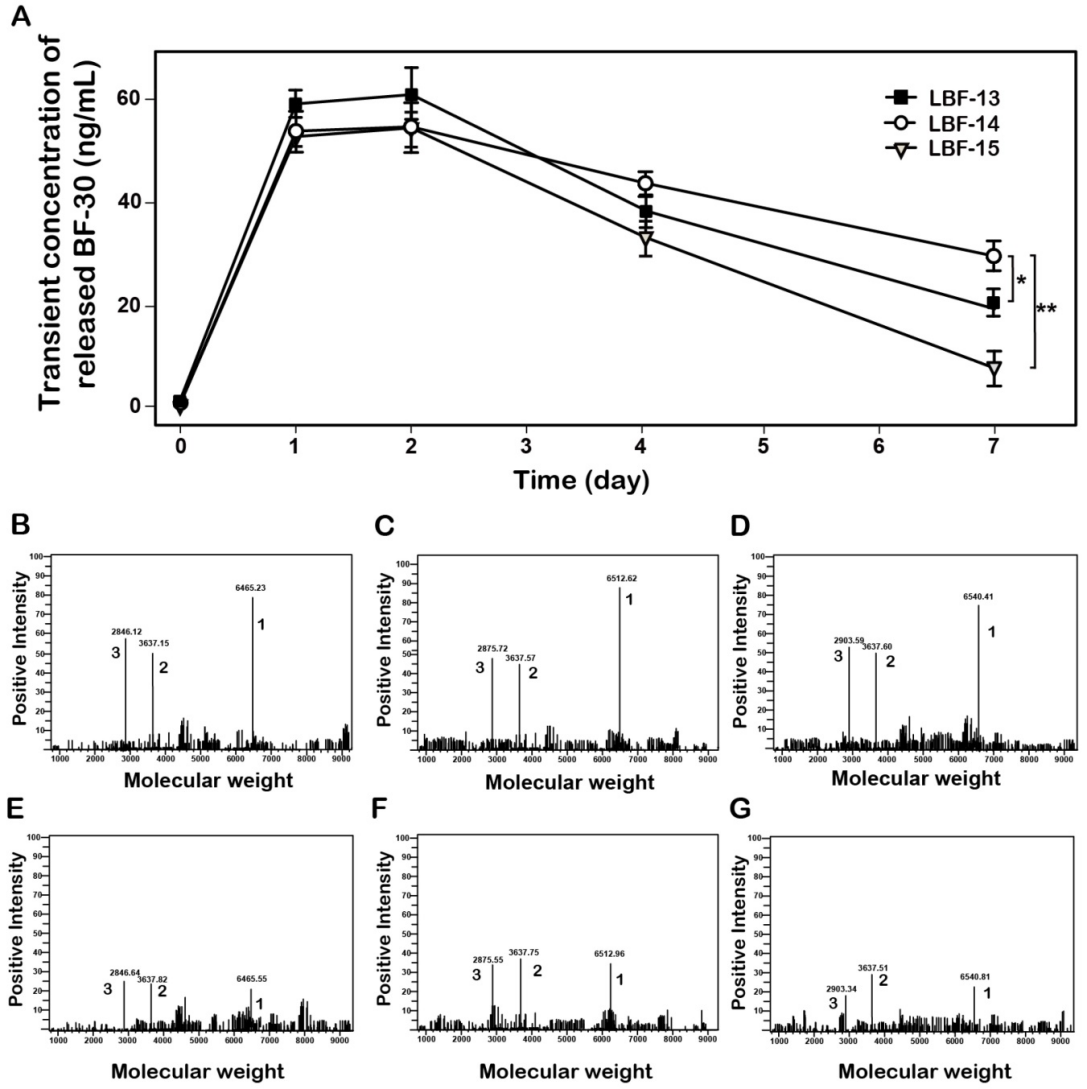
Controlled release of the native BF-30 from three LBF peptides *in vitro*. (A) The transient concentration of the released BF-30-time curve. The mass spectrums of the enzymatic mixtures of LBF-13 at 1 day (B) and 7 day (E), LBF-14 at 1 day (C) and 7 day (F) or LBF-15 at 1 day (D) and 7 day (G) by thrombin. Peak 1, 2 and 3 refer to LBF polypeptides, albumin binding tags and released BF-30, respectively. **P < 0.01 and *P < 0.05. All data are expressed as mean ± SD (n=6).

To confirm whether the thrombin can stably digest and then slowly release the native BF-30 from LBF peptides, the *in vitro* enzymolysis assays in PBS buffer (pH 7.4) containing the thrombin at a final concentration of 0.4 U.ml^-1^ were performed, and the results were shown in Figure 2. As expected, BF-30 was slowly released from all the three fusion peptides via the thrombin cleavage and sustained for about 7 days. Specially, the concentration of released BF-30 in LBF14 group still maintain for above 20 ng/ml at day 7. Moreover, the transient existence of BF-30 in LBF-14 exerted a proper manner with relatively flat rate and prolonged duration of release. Furthermore, the intact LBF peptides, EYEKEYE-(GGGGS)_2_-FNPR, and released BF-30 in the protease cleavage reaction at day 1 and day 7 were further identified by the method of LC-MS/MS (Figures 2B-G).

### *In vitro* plasma stability test

**Figure 3:**
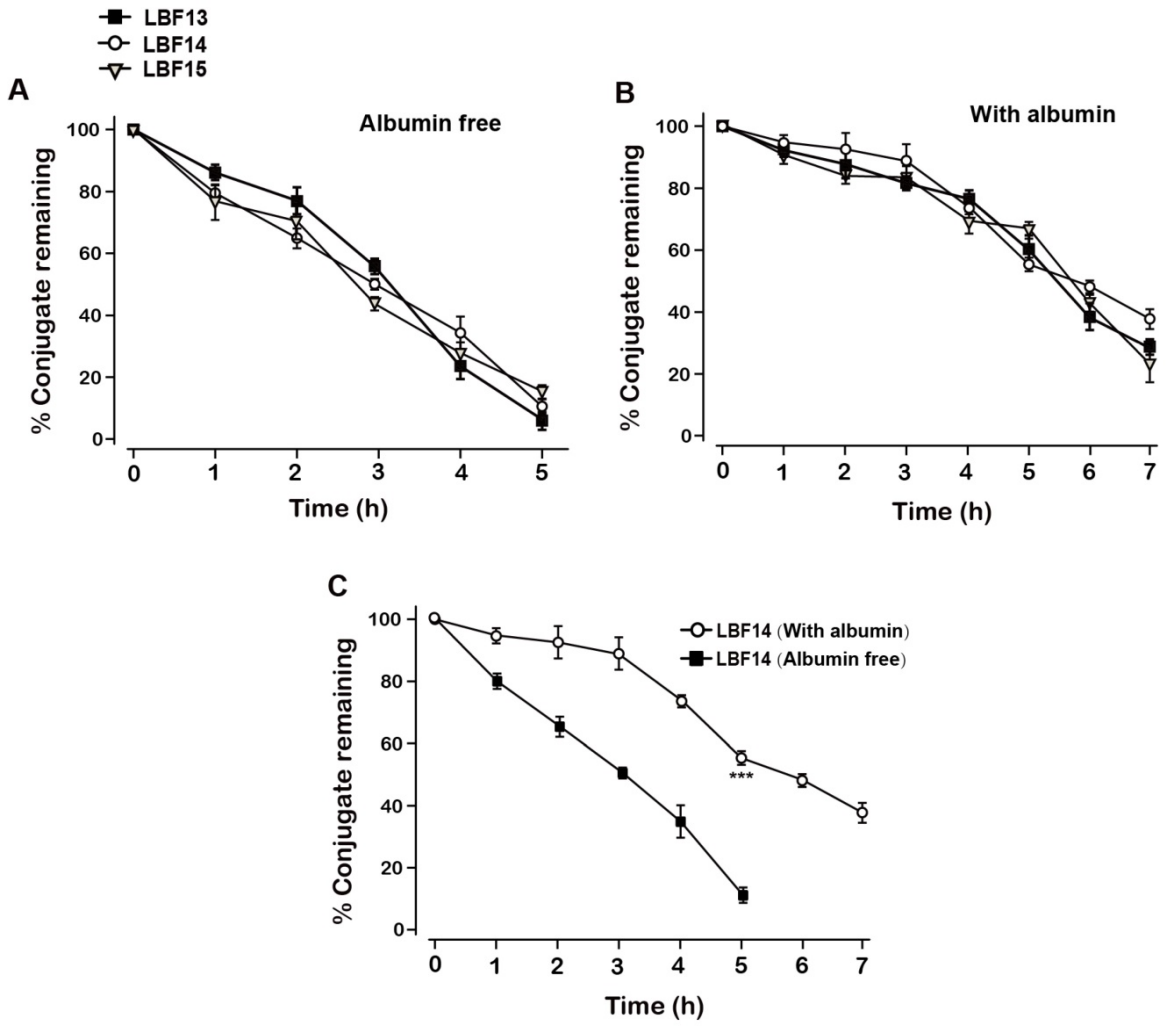
Degradation profiles of LBF-13, LBF-14 and LBF-15 under incubation in plasma without (A) or with (B) serum albumin. (C) %Conjugate remaining of LBF-14 alone without or with albumin. All data are expressed as mean ± SD (n=6). All results were showed as means ± SD (n=6). ***P < 0.001 *vs.* LBF14 group (Albumin free).

The *in vitro* plasma stability tests with or without albumin were conducted to evaluate the plasma stabilities of LBF-13 to LBF-15. As was evidenced in Figure 3A, the residual percent of three LBF peptides were all rapidly decreased in the plasma without albumin and almost dropped to 0% within 5 days. Remarkably, LBF-14 exhibited significantly prolonged half-life (*t*_1/2_~7.5 day) in the mouse plasma (with albumin) compared with LBF-13 and LBF-15 (*t*_1/2_ = 5.5 day and 5.7 day, respectively) (Figure 3B). Furthermore, the %conjugate remaining of LBF-14 on the condition with albumin was significantly higher than that without albumin (Figure 3C). Combined with the results of *in vitro* enzymolysis assay and plasma stability test, LBF-14 was selected for further evaluation of* in vitro* tumour cell inhibitory activity and *in vivo* anti-tumour efficacies.

### Inhibitory activity of the released BF-30 on B16F10 cells

**Figure 4:**
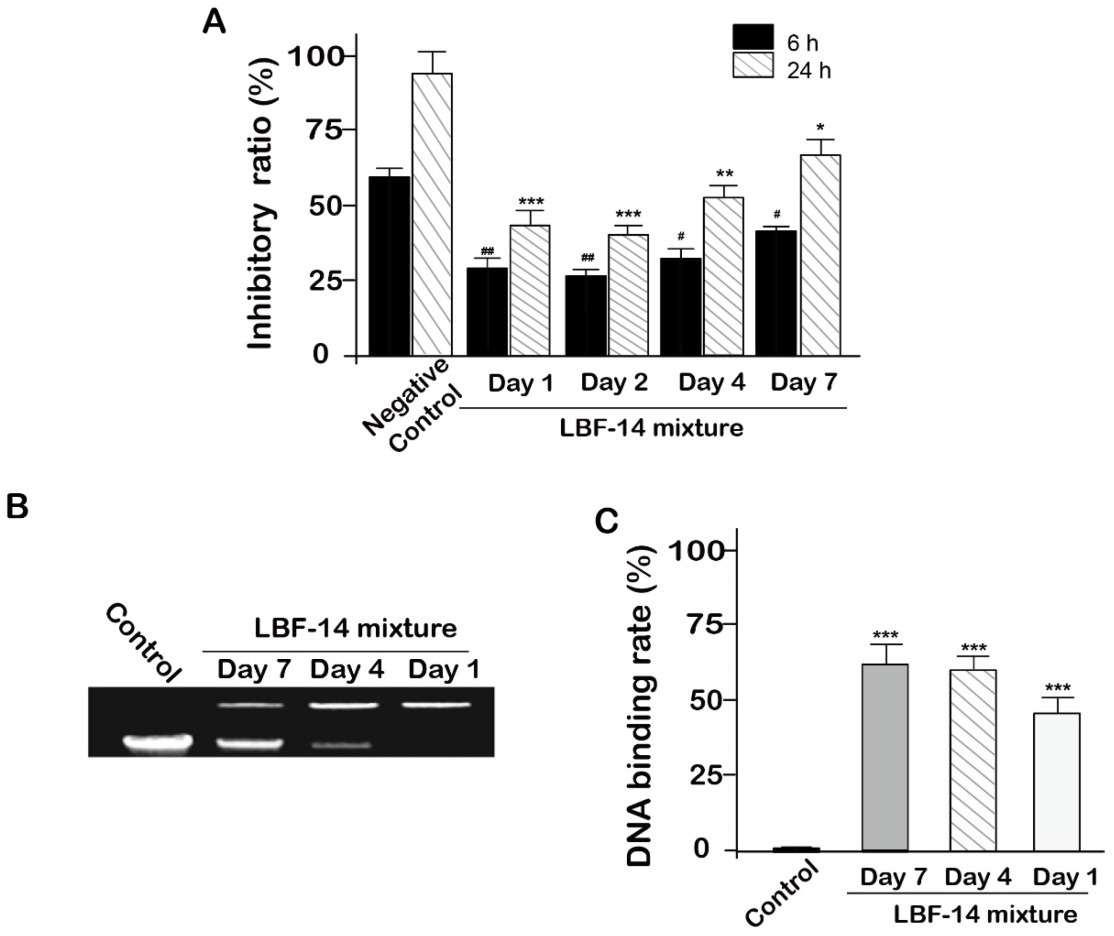
Inhibitory activity of LBF-14 on B16F10 cells. (A) Inhibitory activity of BF-30 released from LBF-14 at day 1, 2, 4 and 7 on the growth of B16F10 cells. The released BF-30 from LBF peptides bound to genomic DNA of the B16F10 cells. (B) Photograph of gel retardation assay of genomic DNA from B16F10 cells treated with the pre-treated LBF peptides; (C) Densitometric analysis of genomic DNA levels. All data are expressed as mean ± SD (n=6). ***P < 0.001, **P < 0.01, *P < 0.05 *vs.* control group.

In order to evaluate the *in vitro* biologic effects of LBF-14, especially for the released BF-30, we accessed the inhibitory activities of four enzymatic mixtures comprising of the released BF-30, albumin binding tag and intact LBF-14 from *in vitro* release test on the B16F10 cells. As was illustrated in Figure 4A, the enzymatic mixtures of day 1, 2, 4 and 7 all significantly inhibited the growth of B16F10 cells with a 24-hour inhibition ratio of 55.8% for day 1, 60.2% for day 2, 48.6% for day 4 and 36.3% for day 7, respectively. The inhibition ratios of the enzymatic mixtures at day 1 and day 2 were slightly higher than that of day 4 and 7, which may be directly related to the transient concentration of the released BF-30 molecules. Moreover, above results proved that our newly designed LBF-14 holds the ability of controlled-release of BF-30 and retained the anti-tumour cells efficacies for at least 7 days.

### DNA retardation assay

The genomic DNA extracted from the B16F10 cells were pre-treated with the released BF-30 from LBF-14 at day 1, 4 and 7 for half an hour. As shown in Figures 4B and 4C, retarded electropheretic migration of released BF-30-treated genomic DNA from B16F10 cells were observed indicating the released BF-30 could bind the genomic DNA. As was illustrated in Figure 4C, densitometric analysis of genomic DNA levels in the gel retardation assay showed that the blocked ratios of the electrophoresis of genomic DNA of B16F10 by released BF-30 at different days were also consistent with the results of inhibitory activities on B16F10 cells.

### Pharmacokinetics study

**Figure 5:**
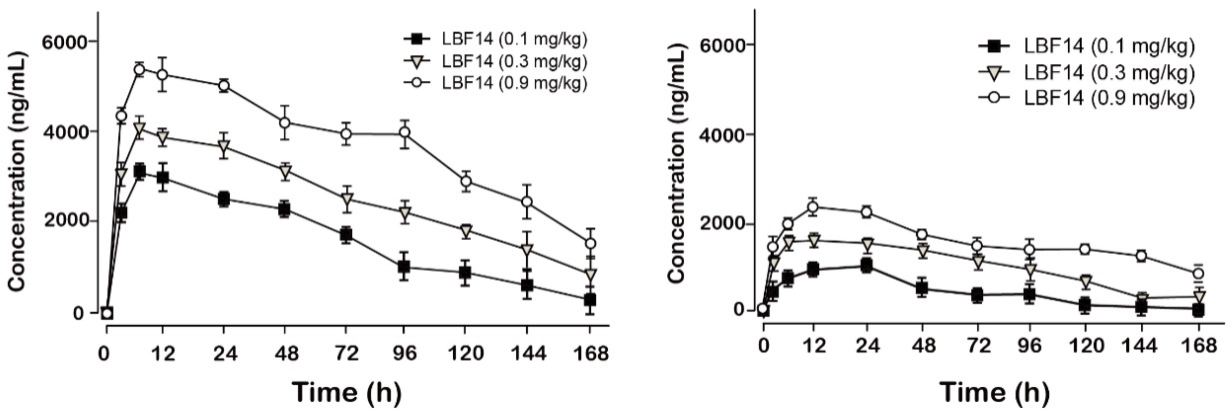
Pharmacokinetic profiles of LBF-14 in healthy cynomolgus monkeys. The plasma concentrations of (A) intact LBF-14 and (B) released BF-30 after the subcutaneous administration of LBF-14 at dose of 0.1, 0.3 and 0.9 mg/kg in monkey plasma. All results were showed as means ± SD (n=5).

Pharmacokinetic tests of LBF-14 at three doses (0.1, 0.3 and 0.9 mg/kg) were performed in healthy cynomolgus monkeys. As was illustrated in Figure 5, LBF-14 at 0.1 mg/kg, 0.3 mg/kg or 0.9 mg/kg held the elimination *t*_1/2_ of 88.1 h, 101.5 h and 120.9 h for intact molecule, and 101.5 h, 107.9 h and 136.4 h for released BF-30, suggesting that LBF-14 has potential to be developed as a weekly anti-tumour agent.

### Chronic anti-tumor efficacies of LBF-14 on B16F10 bearing mice

**Figure 6:**
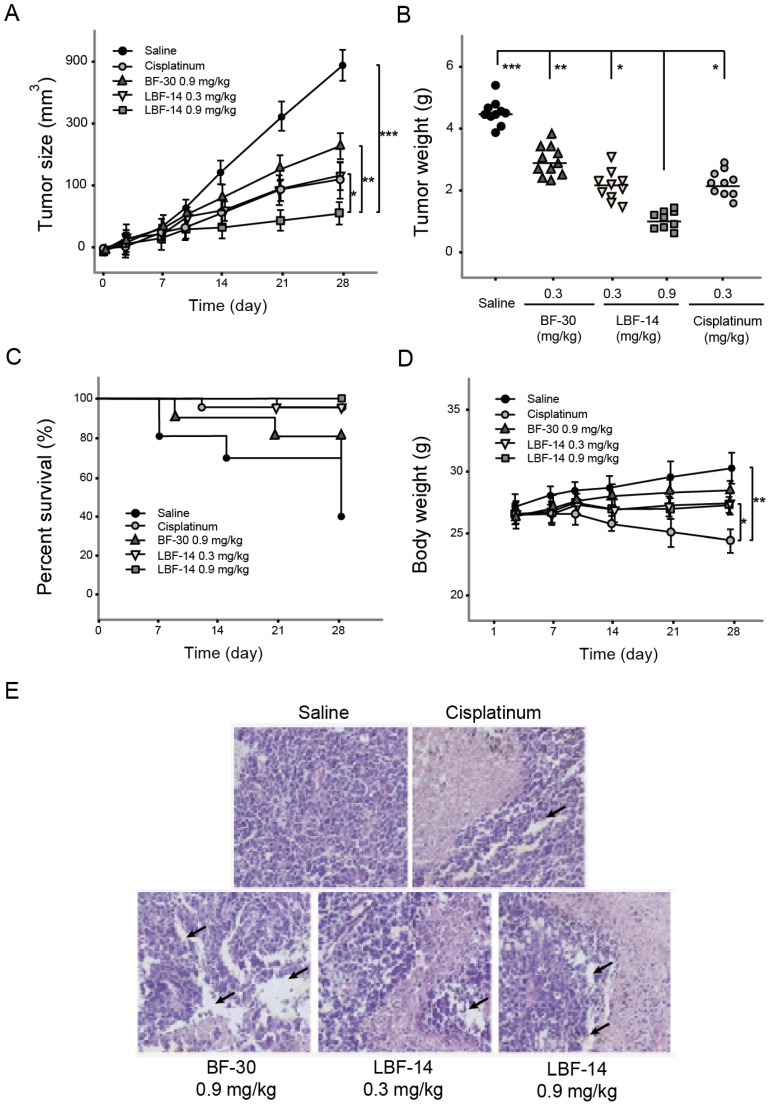
Effective suppression of melanoma growth in B16F10-bearing C57BL/6J mice was induced by 4-week treatment of LBF-14. The (A) tumour size, (B) tumour weight, (C) percent survival, (D) body weight of the B16F10-bearing mice and (E) Pathological images of the tumour tissues of B16F10-bearing mice were accessed. All results were showed as means ± SD (n=10). ***P < 0.001, **P < 0.01, *P < 0.05.

The B16F10 melanoma-bearing mouse model was successfully established by s.c. administration of tumour cells into the C57BL/6J mice to access the *in vivo* anti-tumour activities of LBF-14. The growth rate of tumour size of B16F10 melanoma in model mice was obviously suppressed by the treatment of LBF-14 with inhibitory rates of 63.5% for 0.3 mg/kg and 91.5% for 0.9 mg/kg, respectively, compared with saline treated ones at day 28 (Figure 6A). Similar inhibitory trends were also showed in the change of tumour weight (Figure 6B). As shown in Figure 6C, LBF-14 treated groups exhibited an increased trend of survival rate compared to the negative control mice (100% of LBF-14 at 0.9mg/kg, 90% of LBF-14 at 0.3 mg/kg and 40% of saline). Furthermore, the body weights of LBF-14 treated ones were similar to those of the control ones (Figure 6D), indicating no obvious side effects of LBF-14 on body weight of model mice existed, while the treatment of cisplatinum exerted significantly opposite effects. To further evaluate the anti-tumour effects of three doses of LBF-14 in the B16F10 melanoma-bearing model mice, the tumour tissues of each group were separated and performed the H&E staining. Results of the H&E staining were presented in Figure 6E indicating that the mean tumour cell size of both two LBF-14 treated groups were significantly lower than that of the saline treated group or native BF-30. Moreover, the capsule of the malignant melanoma were almost completely disappeared in the groups treated with BF-30 at the doses of 0.3 and 0.9 mg/kg, which reveal that BF-30 can effectively suppressed metastasis and invasion of B16F10 cells and was comparable to the cisplatinum-treated group.

## Discussion

Metastatic melanoma is a common and most malignant skin malignancy in plastic surgery clinics with high recurrence, and global two hundred thousand new cases as wells as sixty-five thousand deaths every year.^13^, ^14^. According to previously announced statistics, about 20% of patients have metastasis at first diagnosis and the tumour lesions can metastasize to adjacent skin, subcutaneous tissue, regional lymph nodes, and distant organs including the lung, liver, bone, and brain 15. Cisplatinum, as a cell cycle non-specific drug, is a metal complex of inorganic platinum which could inhibit anti-tumour cell growth by disrupting DNA function and preventing DNA replication 16, 17. Cisplatinum holds the wide antitumour spectrum, strong and synergistic effects with multiple anti-tumour drugs and plays an important role in the treatment of advanced or metastatic melanoma 18, 19. However, the side effects of Cisplatinum are also evident, including neurotoxicity, vomiting, nausea, nephrotoxicity, myelosuppression, and weight loss 20-22. In addition to surgical resection and traditional chemotherapy, more and more attention has been paid to biological therapies for the treatment of melanoma without dose-limiting toxicities 23, 24.

In recent years, numerous new researches on antimicrobial peptides have been widely deepened. As an important class of bioactive substances, antimicrobial peptides hold not only antibacterial effects but also anti-tumour activities 3, 25, 26. The anti-tumour mechanism of antimicrobial peptides is more complex than that of antibacterial effects which mainly including two square surfaces: (1) antimicrobial peptides inhibit or kill tumour cells by directly contacting with tumour cells and destroying cell membranes, line microsomes and genomic DNA; (2) antimicrobial peptides recognize and kill tumour cells by actively mobilizing and enhancing the body's immune system 4, 27.

BF-30, a natural antibacterial peptide extracted from the venom of the snake *Bungarus fasciatus*, has shown broad-spectrum antimicrobial activities, especially against drug-resistant bacteria 2. Previous studies have investigated the *in vitro* antitumour activities of BF-30 against murine pancreatic cancer cell line Panc02, and the results showed that the native BF-30 could effectively inhibit the proliferation of Panc02 cells 28. In addition, BF-30 also holds selective killing effects on tumour cells and exhibits non-toxicity to the normal mammalian cells based on the differences in surface charges between the tumour cells and normal ones 1, 3. Anionic molecules, such as phosphatidylserine and O-glycosylated mucins, are overexpressed by most tumour cells, resulting in a negative charge on their cell membrane surface 29, 30. Therefore, the surface of tumour cells or BF-30 present a negative charge or positive charge, respectively, indicating that BF-30 peptides could bind to the membrane of target tumour cells by electrostatic adsorption. On this basis, BF-30 molecules bind to the hydrophobic groups of phospholipids in target cells envelope to form a peptide phospholipid supramolecular complex intercalated into the cell membrane. The hydrophilic part of BF-30 molecule further binds to the envelope lipid to form the transmembrane channel. Subsequently, BF-30 molecules enter the plasma membrane homeotropically, while the original arrangement order of proteins and lipids on the plasma membrane becomes more unstable due to interference by foreign substances. Moreover, BF-30 undergo motor polymerization on the otherwise fragile plasma membrane to form transmembrane ion channels embedded in the plasma membrane, at which time the membrane and plasma membrane of target cells fold anticancer peptide completely breakdown, resulting in spillage of cancer cell contents and loss of a large number of intracellular ions. Not only that, further depolarization of the tumour cell membrane cause the failure of maintaining the intracellular osmotic pressure which leads to the further lysis of the tumour cell membrane or apoptosis^4^. Therefore, the study of BF-30 as an anticancer drug has far-reaching implications. However, the curative effects of BF-30 are restricted in the short duration of action *in vivo* attributed to glomerular filtration and proteolysis, which means that the peptide-based drugs must be frequently administered to maintain the* in vivo* therapeutic level.

HSA, as a widely used* in vivo* drug carrier due to its long lifespan (~19 days) and abundance in blood circulation, might offer more pathways for developing peptide drugs with long-acting and promising therapeutic values 31. According to the previous research 9, the heptapeptide tag (EYEKEYE) with fatty acid chain exerting desired affinity to albumin was preliminarily applied to three bioactive peptides, and the prolonged *in vivo* efficacies were also proved in mouse models.

In this study, we designed twenty-one albumin-binding fusion peptides (termed LBF-1 to LBF-21) by fusing the native BF-30 to twenty-one kinds of heptapeptide tags with aliphatic acids of various lengths at different modification sites via a flexible linker (GGGGS)_2_ and a thrombin-cleavable site (FNPR) (Figure 1). The following albumin-binding affinity measurements revealed that LBF-13 to LBF-15 exert relatively higher binding affinity to albumin compared with other ones (Table 2). Theoretically, better albumin affinity means that BF-30 analogues can bind more tightly to albumin, and the corresponding *in vitro* and *in vivo* stability will be significantly improved. Furthermore, we performed the *in vitro* enzymolysis assay (Figure 2) and plasma stability test (Figure 3), and the results revealed that LBF-14 exhibited the more stably controlled release of native BF-30 and better stability in mouse plasma compared with LBF-13 or LBF-15. Moreover, the concentration of the transient BF-30 in LBF-14 at different time-pionts exerted a proper manner with relatively flat rate and prolonged duration of release (Figure 2). However, the better stability does not represent better in vitro and in vivo efficacies. Therefore, LBF-14 was selected for further evaluation of the *in vitro* inhibitory activity on tumour cells and *in vivo* anti-tumour efficacies.

As is showed in Figure 4A, the released BF-30 in the enzymolysis mixture of the day 1, 2, 4 and 7 all maintained the inhibitory activities on B16F10 tumour cells after both 6 hours and 24 hours of co-incubation, indicating that the LBF-14 holds potential to slowly release the BF-30 and retained the effective *in vitro* anti-tumour cells activities for more than 7 days. Interestingly, the changes of inhibitory ratio of the samples at different time on B16F10 were found to be consistent with change of the exact concentration of released BF-30 showed in Figure 2. According to the previous research, Hui Wang et al. performed similar experiment of DNA retardation and result showed that native BF-30 could bind to genomic DNA of the B16F10 cells and also block the electrophoretic DNA mobility in a dose dependent manner 4. In this study, DNA retardation experiments were performed using the released BF-30 from pre-enzymatic LBF-14 mixture at day 1, 4, and 7. As showed in the photograph of gel retardation assay of genomic DNA from B16F10 cells treated with different enzymatic LBF-30 mixture, the released BF-30 molecules were able to bind genomic DNA in B16F10 (Figure 4B). Densitometric analysis of the relative ratios of genomic DNA levels in the gel retardation assay. The electrophoresis bands were photographed and analyzed using Image software. Moreover, the released BF-30 blocked the electrophoresis of genomic DNA of B16F10 in a dose-dependent manner (Figure 4C).

Therefore, we further proved that LBF-14 exhibited inhibitory effects on B16F10 cells mainly rely on binding the genomic DNA. Not only that, the enzymolysis sample of 7-day also held the ability to realize the effective DNA retardation indicating that the stability of the DNA-binding potency of released BF-30.

The *in vivo* pharmacokinetic parameters of LBF-14 were further evaluated in healthy cynomolgus monkey. As is illustrated in Figure 5,the* t*_1/2_ of intact LBF14 at dose of 0.1 mg/kg, 0.3 mg/kg and 0.9 mg/kg were approximately 88.1 h, 101.5 h and 120.9 h, respectively, and* t*_1/2_ of released BF-30 at dose of 0.1 mg/kg, 0.3 mg/kg and 0.9 mg/kg were approximately 101.5 h, 107.9 h and 136.4 h (Figure 5). In addition, we found that clear dosage-dependency of the transient concentration of LBF-14 in the dosage range of 0.1-0.9 mg/kg existed, which will be beneficial to the future preclinical efficacy study or even clinical application. It is worth discussing that LBF-14 exerted an obviously prolonged *t*_1/2_ in primate animals compared with results in mouse serum indicating that the difference of the affinity between the monkey albumin and mouse albumin probably play important role in the stability differences. The LBF-14 has potential to be developed as a weekly anti-tumour agent based on the pharmacokinetic results.

Considering that the mice are more commonly used in the construction of tumour model, we still used the C57BL/6J mice in the further *in vivo* evaluation of the anti-tumour activities rather than monkeys. As was illustrated in Figure 6, 4-week treatment of LBF-14 at both two doses (0.3 and 0.9 mg/kg) exerted promising anti-tumour efficacies on B16F10-bearing mice *in vivo*. Compared with the saline treated mice, chronic administration of LBF-14 inhibited the tumour size of the B16F10-bearing mice with a dose-dependent inhibition ratio of 63.5% for 0.3 mg/kg and 91.5% for 0.9 mg/kg. In addition, LBF-14 at two doses both obviously increased the percent survival of B16F10-bearing C57BL/6J mice compared with saline treated ones (*P* < 0.05), especially for the high dose administration of LBF-14 at 0.9 mg/kg which maintained a 100% of survival rate (Figure 6C). Moreover, no obvious weight loss of LBF-14 treated mice occurred as showed in Figure 6D, while the cisplatinum treated ones exhibited a significant decrease in body weight. It is worth discussing that after 5 weeks of feeding, the additional backup melanoma model mice in the negative control group showed a cliff decrease in body weight with increasing tumour size (data not supply), suggesting that the health of the body is continuously eroded by tumour cells. Moreover, the body weight of mice in the treatment group also showed a more significant decrease during entire experimental period, which was one of the most significant side effects of these chemical anti-tumour drugs. Furthermore, the anti-tumour effectivities of LBF-14 were further confirmed by histopathological detection and the pathological images revealed that the chronic administration of LBF-14 effectively decreased the vascular network and the number of B16F10 cells in tumour issues (Figure 6E).

An ideal anti-tumour peptide drug should generally have the advantages for desirable efficacy, stability and long residual action, and LBF-14 possesses these advantages precisely. The *in vitro* and *in vivo* stability of LBF-14 were significantly improved by fusion of heptapeptide tags and thrombin-cleavable site. Furthermore, the anti-tumour efficacies of LBF-14 were demonstrated in cell viability assay and the chronic study on B16F10 bearing mice. Compared with traditional chemical drugs and antibody drugs, peptide drugs have the advantages of both low immunogenicity and low cost. Therefore, LBF-14 is expected to be a promising therapeutic candidate for tumours which exerts great antineoplastic activity.

In conclusion, above results revealed that our newly designed BF-30 derivative, LBF-14, contributed to the outstanding anti-melanoma action via disrupting the cytoplasmic membrane of tumour cells, and then binding to the genomic DNA to prevent migration and angiogensis of melanoma cells. Combined with the *in vivo* anti-tumour effects and survival results in B16F10-bearing mice, LBF-14 holds the potential to be developed as an outstanding anti-melanoma drug with low toxicity.

## Figures and Tables

**Table 1:** The HPLC and mass spectra analysis results of BF-30 derivatives.

Peptide	Purity (%)	Molecular mass		Conjugated-Peptide	Purity (%)	Molecular mass	
		Calcaulated	Found			Calcaulated	Found
LBF-1	99.74	[M+6H]^+^ 1008.0	1008.3	LBF-12	98.36	[M+6H]^+^ 1017.7	1017.8
LBF-2	98.37	[M+6H]^+^ 1012.7	1012.5	LBF-13	98.91	[M+6H]^+^ 1008.0	1008.0
LBF-3	99.07	[M+6H]^+^ 1017.7	1017.9	LBF-14	98.67	[M+6H]^+^ 1012.7	1012.8
LBF-4	99.55	[M+6H]^+^ 1008.0	1008.1	LBF-15	99.38	[M+6H]^+^ 1017.7	1017.6
LBF-5	99.76	[M+6H]^+^ 1012.7	1012.4	LBF-16	98.84	[M+6H]^+^ 1008.0	1008.1
LBF-6	99.20	[M+6H]^+^ 1017.7	1017.7	LBF-17	99.10	[M+6H]^+^ 1012.7	1012.9
LBF-7	98.56	[M+6H]^+^ 1008.0	1008.1	LBF-18	99.36	[M+6H]^+^ 1017.7	1017.9
LBF-8	99.47	[M+6H]^+^ 1012.7	1012.8	LBF-19	99.59	[M+6H]^+^ 1008.0	1007.8
LBF-9	99.12	[M+6H]^+^ 1017.7	1017.5	LBF-20	99.35	[M+6H]^+^ 1012.7	1012.8
LBF-10	99.19	[M+6H]^+^ 1008.0	1007.9	LBF-21	99.47	[M+6H]^+^ 1017.7	1017.5
LBF-11	98.69	[M+6H]^+^ 1012.7	1012.6				

**Table 2:** The binding affinity constants of LBF-1 to LBF-21 conjugates for HSA.

Peptide	HSA			Peptide	HSA		
	*k*a (M^-1^s^-1^)	*k*d (s^-1^)	*K*_D_ (M)		*k*a (M^-1^s^-1^)	*k*d (s^-1^)	*K*_D_ (M)
LBF-1	1.95×10^4^	2.43×10^-2^	1.25×10^-6^	LBF-12	7.77×10^3^	3.63×10^-2^	4.67×10^-6^
LBF-2	1.85×10^4^	2.74×10^-2^	1.48×10^-6^	LBF-13	2.51×10^4^	6.11×10^-2^	2.43×10^-7^
LBF-3	1.08×10^4^	3.17×10^-2^	2.94×10^-6^	LBF-14	1.24×10^4^	5.35×10^-3^	4.31×10^-7^
LBF-4	1.51×10^3^	2.51×10^-2^	1.66×10^-5^	LBF-15	2.12×10^4^	6.44×10^-2^	3.04×10^-7^
LBF-5	1.42×10^4^	5.39×10^-2^	3.80×10^-6^	LBF-16	1.01×10^4^	1.54×10^-2^	1.52×10^-6^
LBF-6	8.72×10^3^	6.55×10^-2^	7.51×10^-6^	LBF-17	2.75×10^4^	5.26×10^-2^	1.91×10^-6^
LBF-7	2.03×10^4^	5.88×10^-2^	2.90×10^-6^	LBF-18	2.78×10^4^	4.98×10^-2^	1.79×10^-6^
LBF-8	1.37×10^4^	8.74×10^-2^	6.38×10^-6^	LBF-19	1.58×10^4^	3.47×10^-2^	2.20×10^-6^
LBF-9	2.50×10^4^	6.12×10^-2^	2.45×10^-6^	LBF-20	3.24×10^4^	6.19×10^-2^	1.91×10^-5^
LBF-10	5.67×10^4^	6.54×10^-2^	1.15×10^-6^	LBF-21	1.85×10^4^	3.37×10^-2^	1.82×10^-6^
LBF-11	2.94×10^4^	5.32×10^-2^	1.81×10^-6^				

## Abbreviations

SPR: Surface plasmon resonance
BF-30: Cathelicidin-BF
HSA: Human serum albumin
MTT: Methyl thiazolyl tetrazolium
DMSO: Dimethylsulfoxide
